# The Effect of the Landscape Matrix on the Distribution of Dung and Carrion Beetles in a Fragmented Tropical Rain Forest

**DOI:** 10.1673/031.010.8101

**Published:** 2010-06-29

**Authors:** Alfonso Díaz, Eduardo Galante, Mario E. Favila

**Affiliations:** ^1^ Instituto de Ecología A.C., Departamento de Biodiversidad y Ecología Animal, AP 63, CP 91000, Xalapa, Veracruz, México.; ^2^ Centro Iberoamericano de la Biodiversidad, Instituto de Biodiversidad, Universidad de Alicante, Spain.

**Keywords:** Connectivity, corridors, forest fragmentation, living fences, Los Tuxtlas Biosphere Reserve, Mexico, spatial distribution

## Abstract

Understanding the response of species to anthropogenic landscape modification is essential to design effective conservation programs. Recently, insects have been used in empirical studies to evaluate the impact of habitat modification and landscape fragmentation on biological diversity because they are often affected rapidly by changes in land use. In this study, the use of the landscape matrix by dung and carrion beetles in a fragmented tropical rain forest in the Los Tuxtlas Biosphere Reserve was analyzed. Fragments of tropical rain forest, forest-pasture edges, pastures, isolated trees, living fences (trees connected with barbed wire) and barbed wire fences were studied both near and far from forest fragments. Forest fragments had the highest abundance values, but pastures had the highest dung and carrion beetle biomass. Habitat specificity was high for the beetles in the most dissimilar habitats. Forest fragments and forest-pasture edges had and shared the highest number of species, but they shared only two species with pastures, barbed wire fences and isolated trees. Only one forest species was found within living fences far from the forest fragments. However, approximately 37% of the forest species were caught within living fences near the forest fragments. Therefore, forest-pasture edges function as hard edges and prevent movement among forest fragments, but living fences seem to act as continuous habitat corridors when connected to forest fragments, allowing forest beetles to move between the fragments. Further studies are necessary to determine the minimum width of living fences necessary to provide good corridors for these beetles and other species.

## Introduction

Tropical forest fragmentation has led to a radical modification of the landscape and has had a huge impact on biodiversity (Doherty and Grubb 2002; Wade et al. 2003). More than one-third of species disappear when habitats are fragmented (Klein 1989; Driscoll 2004) and many species become extinct in small patches (Thomas 2000; Lindenmayer et al. 2002). Pastures, agriculture, and landscapes with a mixture of management strategies occupy approximately 70% of the land in the tropics (McNeely and Scherr 2003). As most tropical ecosystems are fragmented, the conservation of biodiversity should not only consider the protected areas, but also the managed ecosystems (Perfecta and Vandermeer 2008).

In tropical agricultural ecosystems it has been shown that a high proportion of species also survives in the remnant patches of original vegetation because they are able to use the landscape matrix (Andren 1994; Didham et al. 1998; Vandermeer and Perfecto 2007; Perfecto and Vandermeer 2008). The matrix may affect the rate of movement of organisms among tropical forest patches and thus influence extinction rates on a regional level (Gustafson and Gardner 1996; Perfecto and Vandermeer 2002). Forest species generally require connections to move between fragments in anthropogenic landscapes (Noss 1991; Soulé and Gilipin 1991), but the movement of forest species may be limited by their sensitivity to edges and by matrix quality (Murcia 1995; Magura et al. 2001; Perfecto and Vandermeer 2002; Meyer et al. 2008). The quality of the matrix in agricultural landscapes is becoming an important component in the conservation of tropical ecosystems (Gascon et al. 2000; Perfecto and Vandermeer 2008). Since pastures are one of the most common managed ecosystems in the tropics (McNeely and Scherr 2003), it is also necessary to know how forest species respond to the matrix in pasture landscapes before one can decide how to best maintain connectivity among the remaining fragments.

Insects play important ecological roles in diverse ecological processes such as nutrient cycling, seed dispersal, bioturbation, and pollination (Nichols et al. 2008). However, knowledge about the response of insects to human activity is limited compared to that for other taxa (McGarigal and Cushman 2002). The dung beetles of the subfamily Scarabaeinae (Coleoptera: Scarabaeidae) are a group of insects that are abundant in tropical regions (Halffter and Favila 1993) and participate in some key ecological processes (Estrada and Coates-Estrada 1991; Andresen and Feer 2005; Nichols et al. 2008). These two combined proprieties (abundance and ecological role) led to their being successfully proposed as a biological indicator insect group that can be used to analyze the effect of tropical forest fragmentation on biodiversity and niche structure modification (Halffter and Favila 1993; Favila and Halffter 1997; Spector 2006).

In this paper, the effect of landscape matrix quality on dung beetle composition, distribution and abundance in a tropical fragmented landscape where pastures are the dominant managed ecosystem was analyzed. Six anthropogenic habitats were selected from the tropical landscape: forest fragments, forest-pasture edges, pastures, living and wire fences, and isolated trees. It was predicted that, in this landscape, the changes in vegetation structure from forest to pasture would lead to a reduction in the species diversity of forest carrion and dung beetle assemblages and would result in changes in their guild structure. It was also predicted that living fences act as continuous habitat corridors for dung beetles leaving the forest, while isolated trees in pastures could serve as stepping stone habitats allowing forest beetles to cross pastures in search of other forest fragments. In contrast, pastures were expected to represent barriers.

## Materials and Methods

### Study area

Los Tuxtlas is the northernmost remnant of Neotropical rain forest (Dirzo 1992). In 1998, this area was declared a Biosphere Reserve by the Mexican government (Diario Oficial 1998). The total protected area is 155,122 ha, and the nucleus conservation zone is located on the tops of each of the three main mountains in the reserve: San Martín Tuxtla (1,720 MASL) with 9,806 ha of protected area, Santa Marta (1,660 MASL), with 18,032 ha protected, and San Martin Pajapan (1,245 MASL) with 1,883 ha protected. The land surrounding these mountains makes up the buffer zone (125,401 ha). Of the entire protected area, 19.16% falls within the nucleus, and the remaining 80.84% falls in the anthropic landscape where cattle pastures, crops, and forest fragments predominate (Guevara et al. 2004). This landscape generates environmental conditions that differ radically from those of the original, continuous forest. In Los Tuxtlas, most of the forest fragments are isolated, but some are connected by living fences that delimit the areas used by cattle. Living fences are made with *Bursera simaruba* (Sapindales: Burseraceae), *Erythrina folkersii* (Fabales: Fabaceae) or *Gliricidia sepium* (Fabales: Fabaceae) trees. Planted very close together and connected with barbed wire, they tend to border forest fragments (Guevara et al. 1998). Living fences generate particular environmental conditions with respect to light intensity, temperature, and humidity, and this may allow some forest species to move through them between forest fragments. Larger pastures also have isolated trees that have not been cut down by farmers (Guevara et al. 1998). These are usually forest species such as *B. simaruba, Zanthoxylum kellermanii* (Sapindales: Rutaceae), *Nectandra ambigens* (Laurales: Lauraceae), and *Ficus yoponensis* (Rosales: Moraceae), which provide shade for cattle. These isolated trees might act as stepping stone habitats for some forest species. In Los Tuxtlas, the use of isolated trees and living fences by birds and bats is well documented (Estrada et al. 2000; Estrada and Coates-Estrada 2001). However, the use of these habitats by other taxa is almost unknown. Estrada et al. (1998) suggest that living fences can act as stepping stone corridors for dung beetles in agricultural landscapes, although, by definition, a stepping stone habitat would be better represented by isolated forest fragments and isolated trees.

Fieldwork was conducted on the southwestern slope of the San Martin Volcano (18° 29′ 04″ N, 95° 13′ 28″ W) at an altitude of 600 MASL. This area lies within the buffer zone of the Los Tuxtlas Biosphere Reserve in Veracruz, Mexico (Guevara et al. 2004). Mean annual temperature is 26° C, and mean annual rainfall is 4500 mm. The dry season is from March to May with 11.7 mm of rain per month, and the rainy season is between June and February with a mean monthly rainfall of 486.25 mm (Soto 2004). The selected area covers 8.5 km^2^ and contains two forest fragments (40 and 137 ha). The inner portion of each forest fragment and the forest-pasture edges were sampled. The pastures adjacent to each forest fragment were sampled the same way. Additionally, two living fences, four isolated trees in the pastures, and two barbed wire fences as a control were sampled near and 4 km away from the fragments. Living and wire fences near forest were connected to the fragments. Pastures away from the fragments were not sampled because M. Favila (unpublished data) found no significant differences in dung beetle composition in this managed ecosystem in the buffer zone of the reserve.

The most abundant tree species in the two forest fragments selected were *Esembequia* sp. (*n* = 89 individuals), *Psicotria souzae* (*n* = 96), *Psicotria limonensis* (*n* = 50), *Pinus chiapensis* (*n* = 21), *Casearia nitida* (*n* = 53), *Croton shiedeanus* (*n* = 86), and *Ocotea* sp. (*n*= 130). The living fences consisted of the tree species *B. simaruba* (diameter breast height > 25 cm; height = 10–15 m) (*n* = 18), *Ficus* sp. (diameter breast height > 25 cm; height = 10–15 m) (*n* = 2), *Ocotea* sp. (*n* = 51), *C*. *shiedeanus* (*n* = 41), *E. folkersii* (*n* = 20), *Abarema idiopoda* (diameter breast height > 25 cm; height = 10–15 m) (*n* = 3), *Cestrum oblongifolium* (*n* = 11), *Acacia cornigera* (*n* = 3), *Z. kellermanii* (*n* = 2), *Piper* sp. (*n* = 1), *Trichilia hirta* (*n* = 8), *G. sepium* (*n* = 1), *Eugenia acapulcensis* (*n* = 2), *Tabernaemontana alba* (*n* = 10) and *Psidium guajava* (*n* = 1). Active pastures with cows and horses were characterized by *Conostegia xalapensis, Solanum rudepalum*, *Melampodium divaricatum, A. cornigera, Mimosa pudica, Desmodium* spp., *G. sepium,* and *Sida rhombifolia.* The isolated trees in the pastures were *Mangifera indica* (*n* = 2), *Pouteria sapota* (*n* = 2), *Citrus limon* (*n* = 6), *Cedrela odorata* (*n* = 6), *Persea chiedeana* (*n* = 4), and *Pimienta dioica* (*n* = 8). All of the isolated trees in the pastures were introduced species (diameter breast height > 10 cm; height = 6 – 20 m). Trees were sampled using two 1 × 10 m plots in forest fragments, edges, and living fences. In pastures, sampling plots were 100 × 100 m. Pasture vegetation was sampled using two 1 × 1 m plots.

### Sampling design

Dung and carrion beetles were sampled at each study site with a widely used method: baited pitfall traps arranged along transects (Halffter and Favila 1993; Spector and Ayzama 2003). The traps, plastic pots 12 cm in diameter and 8 cm deep (type A in Halffter and Favila 1993), were arranged along two linear transects spaced 500 m apart. Inside the forest fragment, at the forest-pasture edge, and in the pasture eight traps were used per transect. Each trap was separated by 20 m, but the bait type (40 g human dung or 40 g fish carrion) was alternated so the distance between traps with the same type of bait in each transect was 40 m. After this sampling design had been implemented, Larsen and Forsyth (2005) recommended a minimum distance of 50 m between traps to avoid between-trap interaction in the attractiveness of the different types of bait. In future designs it would probably be prudent to use Larsen's distance, even though in their study they show trap interaction only for one species and in a particular semi-deciduous tropical forest. More studies are required to define minimum distance between traps to avoid bait interaction, and it should be taken into account that this distance may vary for each species, for the type of habitat analyzed, and for different regions.

Traps inside the fragments and in the pastures were set 200 m into and away from the forest edge, respectively. For the forest-pasture edges, four traps were placed one meter into the forest, and the other four were placed one meter away from the outside edge. Additionally, for the living and barbed wire fences near and away from the forest fragments, eight traps at each site were set in the soil 20 meters apart with alternating bait type (human dung or fish). At the isolated trees, four traps (two baited with human dung and two with fish) were spaced out beneath the canopy. The traps were set monthly for six days and six nights and were baited every 48 h at 10:00. Fieldwork was conducted monthly during one week in the middle of the wet season between July and September in 2001 and 2002 in order to obtain an adequate representation of the dung beetle community. A total of 144 traps were used.

Captured dung and carrion beetles were identified to the species level and counted. Voucher specimens of the beetles were deposited in the collection of the Department of Biodiversity and Animal Ecology, Instituto de Ecología, A.C. and in the entomological collection CEUA of the Centro Iberoamericano de la Biodiversidad, University of Alicante, Spain.

### Analyses

The species richness data was pooled for each habitat and compared among study sites with the Mao-Tau sample-based rarefaction curve in Estimates program 8 (Colwell 2006). This moment-based function allows for the direct comparison of sample-based rarefaction curves for different sample sets along with their 95% confidence intervals (Colwell et al. 2004). The sampling unit was an individual trap over a 48 h period, but the values were rescaled by the cumulative number of individuals in order to make the comparison in terms of species richness (Gotelli and Colwell 2001).

Dominance-diversity curves, based on the number of individuals per species, were used to compare the changes in community structure for each habitat. Changes in the community composition of dung and carrion beetles in the different habitats were analyzed by: a) food relocation method: proportion of burrowers to rollers; b) food preference: proportion of generalists (species for which less than 80% of total individuals were caught in copro- or necro-traps) to specialists (species in which more than 80% of the individuals were collected in either copro- or necro-traps); c) diel activity: the proportion of nocturnal to diurnal species; and d) beetle size: large (≥ 10 mm in length) or small (< 10 mm). Diel activity was assigned for each species using data from Favila and Díaz (1997) and Favila (2005). The R × C test of independence using the *G*-test was performed to compare the proportion of individuals in each functional group per habitat. (Sokal and Rohlf 1981).

The biomass of individuals of each species was obtained by drying 20 beetles at 120° C for 48 h, after which the individuals were weighed to the nearest mg and an average individual mass for each species was obtained. The total biomass of the beetles caught in each trap was calculated for each habitat by multiplying the abundance of each species present in a trap by the average biomass per individual for that species and adding the resulting values. The average biomass per individual beetle was calculated for each habitat by multiplying the abundance of each species by its average individual mass, adding across all species, and dividing by the total number of individuals (Spector and Ayzama 2003). A one-way analysis of variance (ANOVA) was used to examine pertrap abundance, per-trap biomass, and the average mass of an individual beetle among the habitats. Significant differences were identified using Tukey's Studentized Range test.

Changes in species composition between habitats were analyzed using a cluster method. The abundance matrix data was first subjected to a square root transformation and standardization. Then it was analyzed using Group Average Linking with a Bray-Curtis similarity matrix. Also, to assess the similarity of dung beetle communities across habitats, Sørensen's classic similarity index (*S*_clas_), which is based on species incidence, was compared with a new statistical approach using abundance-based data, Sørensen's abundance estimator (*S*_abd_). This new test takes into account not only the relationships between the two sites based on their shared species, but also the species that are potentially shared (unseen species) between both sites even though they were not present in the samples (Chao et al. 2005). The analysis was carried out using the Species Prediction and Diversity Estimation program (Chao and Shen 2003).

## Results

Over both periods, 1493 beetles belonging to 30 species were caught in the baited traps (Table 1). Dung beetle species belonged to Scarabaeinae, Aphodiinae, and Hybosorinae. Carrion beetles belonged to Scarabaeinae, Silphidae, and Trogidae. The Scarabaeinae were clearly more abundant (1372 individuals belonging to 23 species) than were the Aphodiinae (7 individuals and 3 species), Silphidae (9 individuals and 1 species), or Trogidae (4 individuals and 1 species). Hybosorinae (101 individuals and 2 species) is a coprophilous group and was excluded from the analysis.

Forest fragments and their edges had the highest number of species (16 and 17, respectively). Two species collected in the edges, *Coprophanaeus pluto* and *Dichotomius colonicus*, were abundant in the pastures (Table 1). In pastures, 10 species were collected from open areas, but two of them were mostly caught in the forest fragments, *Copris sallei* and *Dichotomius satanas.* Thirteen species were caught under the living fences near the forest fragments, and six of these were mostly caught in forest fragments. Of the four species caught under living fences away from the forest fragments, only one belonged to the forest. Nine and 10 species were collected under the barbed wire fences near and away from the forest fragments, respectively. Only one species from the forest, *Ataenius cribrithorax*, was collected beneath the barbed wire fences away from the forest fragments. Beneath the isolated trees near and away from forest fragments, five species were caught; two of these were mostly found in the forest fragments, *D. satanas* and *Uroxys microcularis*, and only very infrequently beneath the trees.

**Figure 1.**
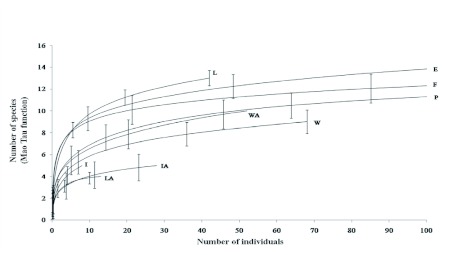
Expected species accumulation curves based on the Mao-Tau function for all habitats. The sampling unit (48 h/trap) was rescaled by the cumulative number of individuals. F = Forest fragment, E = forest-pasture edge, P = pasture. Near fragments: L = living fence, I = isolated tree, W = wire fence. Four km away from fragments: LA = living fence far away, IA = isolated tree far away, WA = wire fence far away. High quality figures are available online.

**Figure 2.**
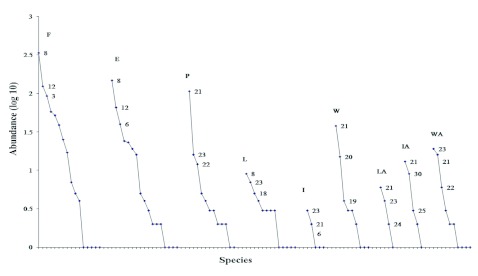
Dominance diversity-curves based on the number of individuals (log10 abundance) per species in each habitat. Order of species importance. F = forest fragments, 8 = *Canthidium centrale,* 12 = *Uroxys microcularis*, 3 = *Deltochilum pseudoparile*; E = forest-pasture edges, 8 = *C. centrale,* 12 = *U. microcularis*, 6 = *Dichotomius satanas*; P = pastures, 21 = *Coprophanaeus pluto*, 23 = *Dichotomius* *colonicus*, 22 = *Copris lugubris.* Close fragments: L = living fences, 8 = *C*. *centrale,* 23 = *D*. *colonicus*, 18 = *Phanaeus endymion*; I = isolated trees, 23 = *D*. *colonicus,* 21 = C. *pluto,* 6 = *D*. *satanas*; W = wire fences, 21 = *C*. *pluto*, 20 = *Puto* *mexicanus*, 19 = *Phanaeus tridens.* Four km away from fragments: LA = living fences far away, 21 = *C*. *pluto*, 23 = *D*. *colonicus,* 24 = *Scatimus ovatus*; IA = isolated trees far away, 21 = *C*. *pluto*, 30 = *Oxelytrum discicolle*, 25 = *Onthophagus batesi*; WA = barbed wire fences far away, 23 = *D*. *colonicus*, 21 = *C*. *pluto,* 22 = *C*. *lugubris.* High quality figures are available.

According to the Mau-Tau function, the number of species is similar in the forest fragments, the edges, the pastures, the living fences near fragments, and some wire fences. Living fences away from the forest fragments, isolated trees, and one wire fence had the lowest number of species (Figure 1). The dominance-diversity curves revealed differences in the order of species importance among the habitats analyzed (Figure 2). In the forest fragments and in forest-pasture edges, the dominant species were *Canthidium centrale* and *U. microcularis.* The next most abundant species were *Deltochilum pseudoparile* and *D. satanas*, all of which are considered forest species (Favila and Díaz 1997; Favila 2005). Under the living fences near the forest fragments, *C*. *centrale* (forest species) and *D. colonicus* (pasture species), were the dominant species; *P. endymion* and *O. batesi* (both pasture species), were the next most abundant species. In pastures, under living and barbed wire fences away from fragments, the dominant species were *C*. *pluto*, *D. colonicus, Scatimus ovatus* and *Copris lugubris,* all common to open areas in the Mexican tropics (Halffter et al. 1992). Under isolated trees near and away from fragments, *C. pluto, D. colonicus, O. batesi* and *Oxelytrum discicolle* were the dominant species from open habitats, and *D. satanas* was the only species from the forest. All the dominance-diversity curves showed a similar steep slope.

**Table 1.**
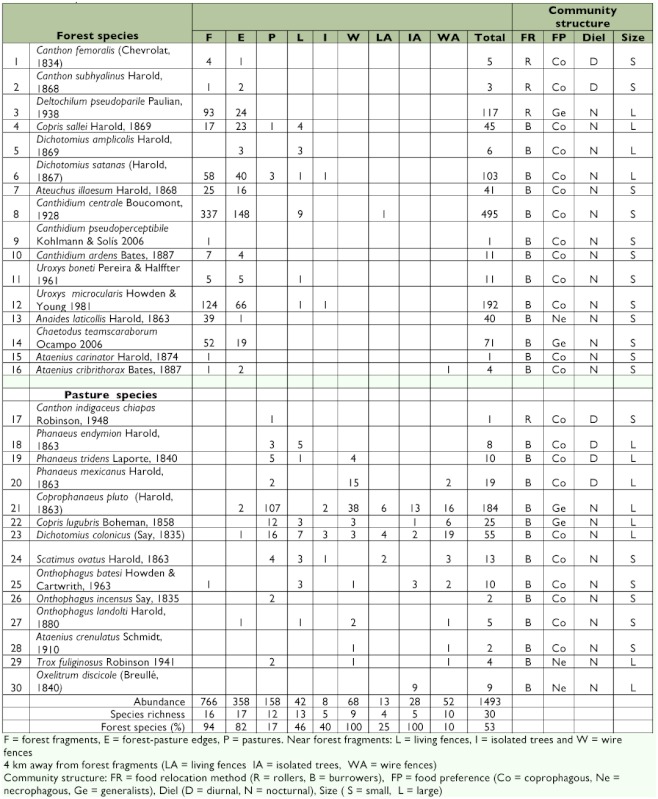
Pooled abundance and richness data for dung and carrion beetles caught in different habitats in a fragmented landscape of the Los Tuxtlas tropical rain forest.

Changes were found in the assemblage structure of dung beetles. Nocturnal individuals were more frequent in fragments, edges, both living and wire fences away from the fragments, and isolated trees both near and away from the fragments (*G* = 21.56, df = 8, p = 0.0057). Pastures, living fences and barbed wire fences near the forest fragments had fewer individuals of nocturnal species. Ball roller beetles were found in one fragment and its edge (*G* = 21.18, df = 8, p = 0.0066). The other habitats had predominantly burrower beetles. Coprophages were more frequently found in living fences near forest fragment and edges (*G* = 16.89, df = 8, p = 0.0311). The other habitats had a mixture of coprophagous and necrophagous beetles. Big beetles (> 10.0 mm) were mostly found in pastures, but small beetles were mostly found in forest fragments, edges and under living fences near fragments (*G* = 15.60, df = 8, p =0.0484).

The abundance of dung beetles per trap differed significantly among habitats (*F* = 27.9, df = 8, p < 0.001). Forest fragments and forest/pasture edges had the highest abundance values (51.3 and 23.9 of total abundance, respectively; Tukey p < 0.05).

Biomass per trap was also significantly different among habitats (*F* = 6.29, df = 8, p = 0.006). The pastures and barbed wire fences had the highest biomass values (27.9 and 31.9%) of total biomass) and these were statistically different from the biomass under isolated trees near fragments and living fences far from fragments (Tukey p < 0.05). Similarly, the average biomass per individual beetle was significantly different among habitats (*F* = 42.9, df = 8, p = 0.01). Once again, the pastures and barbed wire fences also had the highest average biomass values (28.1 and 28.9% of average biomass, respectively; Tukey p < 0.001).

**Figure 3.**
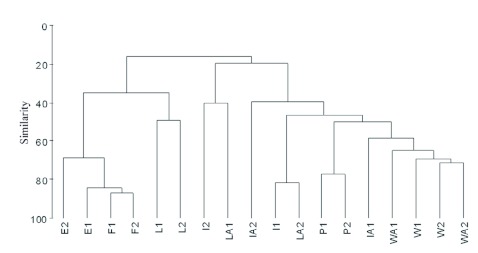
Cluster analysis of habitats with abundance matrix data, using the Bray-Curtis similarity coefficient and group average linking as the clustering method. F1–F2 = forest fragments, E1–E2 = forest-pasture edges, P1–P2 = pastures. Near fragments: L1–L2 = living fences, I1–I2 = isolated trees, and W1–W2 = wire fences. Four km away from fragments: LA1–LA2 = living fences away, IA1–IA2 = isolated trees away, and WA1–WA2 = wire fences away. High quality figures are available online.

Cluster analysis of the habitats using the abundance matrix data revealed two groups (Figure 3). One group was made up of the forest fragments with their respective edges linked to living fences near forest fragments. The other group included a subgroup formed by pastures, all the barbed wire fences, and one isolated tree away the fragments; this subgroup was linked to one living fence away from forest fragments, one isolated tree near the fragments and another isolated tree away from the forest fragments. Also, an isolated tree near the forest fragment and a living fence away from the forest fragments were linked to them.

The classic Sørensen index and the abundance-based Sørensen estimator, adjusted for unseen species, showed that the forest and fragment edges were most similar. Forests and pastures, wire fences, living fences away from forest fragments, and isolated trees were the least similar. However, the similarity between forests and living fences near the fragments was higher than either of those habitats relative to open areas (Table 2).

The abundance-based Sørensen estimator clearly showed greater similarity in composition between the forest and the other habitats, with the exception of the pastures and wire fences. The similarity between forest fragments and living fences near the fragments, when unseen species were considered, was notably greater than the similarity given by the classic Sørensen index.

**Table 2.**
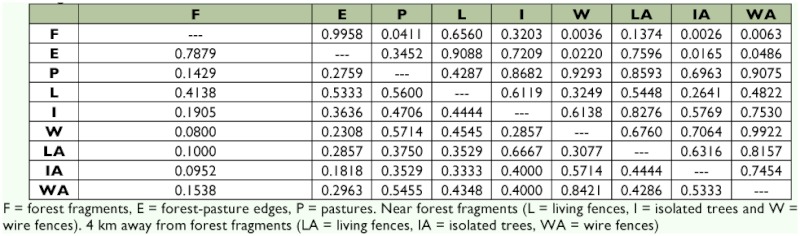
Compositional Sørensen index matrix (above: abundance-based, below: incidence-based) between forest fragments versus eight habitats

## Discussion

Changes in vegetation structure from forest to pasture in the fragmented landscape located in the buffer zone of the Los Tuxtlas Biosphere Reserve have led to a reduction in the species diversity of forest dung and carrion beetles. Of the 16 species found in the forest fragments and their edges, only two were found in pastures. Thus, 87% of the forest species are unable to leave the forest fragments. Similar results have been found inthe tropical forest of Palenque (Mexico) by Halffter et al. (1992) and in other fragmented Neotropical forests (Howden and Nealis 1975; Klein 1989; Scheffler 2005). The high diversity and abundance of the beetles that are trapped in the forest fragments, compared with the other habitats, suggests a “pressure cooker” effect with unknown consequences for the populations. If forest species cannot leave the isolated fragments they could eventually exhaust their resources and have local extinctions by this or by other causes, but that remains to be studied.

The anthropogenic disturbances in forest fragments severely affected dung beetle communities, increasing the isolation of populations and negatively affecting ecological processes (Klein 1989; Halffter et al. 1992, Nichols et al. 2008). Species evenness in communities in undisturbed habitats is usually greater than that of disturbed habitats, and this is the reason the dominance-diversity curves have much less pronounced slopes (Feinsinger 2003). The fact that all the dominance-diversity curves in the habitat studied have steep slopes, suggested that the fragments acted like disturbed habitats, affecting the dung beetle community structure. The most notable change in community structure was the reduction in the number of ball roller species. In the undisturbed forest habitats of the Los Tuxtlas Biological Research Station, UNAM, Favila and Díaz (1997) found nine roller species, but only three rollers were found in the forest fragments analyzed in this study. The absence of 66% of the forest roller species in the remnant patches of forest has been associated with the loss of mammalian fauna in the Los Tuxtlas region (Estrada and Coates-Estrada 2002), but other factors also could have affected the distribution of these species. The habitats studied are at 600 MASL, while the altitude of the Los Tuxtlas Biological Research Station, UNAM ranges from 150 to 300 MASL. This difference in altitude could generate variations in climate and affect the natural distribution of some species. For example, *Canthon indigaceus chiapas*, a species typical of open areas in the Mexican tropics, was very abundant at lower altitudes in the Los Tuxtlas Biological Station (Favila and Díaz 1997) but was scarce during this study (see Table 1). Not only that, the number of species in these forest patches was 34% lower than the number of species found in the intact, continuous forest, i.e. the nucleus areas of the Los Tuxtlas Reserve (Favila and Díaz 1997; Estrada et al. 1998; Estrada and Coates-Estrada 2002; Favila 2005).

In the tropical rain forests of Brazil, Bolivia, and Mexico, dung beetles decrease in size from the forest to pasture or toward natural savanna (Klein 1989; Halffter et al. 1992; Spector and Ayzama 2003; Scheffler 2005). Contrary to the findings of those studies, large necrophagous species dominated in pastures, isolated trees, wire and living fences. Even though this might represent a stochastic event, it would be very interesting to compare the size of dung beetles in other forest fragments and analyze whether this change is related to any modification of the environment. Furthermore, the fact that beetle biomass per trap and biomass per individual beetle were higher in pastures than in the forest habitats is related to the greater abundance of trophic resources (i.e. cattle dung and carrion) in these treeless habitats (A. Díaz, unpublished data). These findings contrast with those of Spector and Ayzama (2003), who found that biomass decreases from forest to edge and from edge to pasture.

New paradigms in conservation biology propose that connections among patches of natural habitat via corridors should be analyzed in the context of the agricultural or managed matrix. The objective is to have a matrix that is “biodiversity friendly” and facilitates the movement of forest species among forest patches (Perfecto and Vandermeer 2008). The high turnover of species between pastures and forest fragments (between 88 and 100%) shows that few forest species colonize the pastures (*C*. *sallei* and *D. satanas*). Clearly, there is a strong edge effect between forest and pasture, one that is not particularly “biodiversity friendly.” Similar results have been found by Spector and Ayzama (2003) in their comparison of dung beetle diversity in forests and natural savannas and their evaluation of the edge effect between both habitats. So, for dung beetles, these represent “hard edges” that do not allow movement among forest fragments via pastures. Hence, the conservation of the beetle species that are affected by habitat boundary could be partially achieved by actively promoting re-growth vegetation along forest edges. This would mitigate the edge effect and decrease the isolation of the fragment in the matrix.

Connections between natural areas and managed landscapes can help to sustain biodiversity and the natural processes of ecosystems and to transform the unfriendly matrix into a friendlier managed matrix (Bennet 2003, Vandermeer and Perfecto 2007, Perfecto and Vandermeer 2008). These findings, and those of Estrada et al. (1998), indicate that living fences act as continuous habitat corridors and allow forest beetles to colonize other forest fragments. This study found 37% of the forest dung beetle species under living fences connected to fragments, and the dominant species were coprophages from the pastures (*D. colonicus*) and the forest (*C*. *centrale*). This indicates that these habitats behave as a membrane that can be crossed by both forest and pasture species. The Sørensen similarity index revealed that the habitats most closely related to forest fragments, with the exception of forest/pasture edges, were the living fences near forest fragments. So, the prediction that living fences act as continuous habitat corridors for dung beetles leaving the forest is valid only for living fences that are connected to forest fragments, not for living fences that are far from them. However, isolated trees had very few forest species and their abundance was low. Isolated trees cannot, therefore, be considered good stepping stone habitats that offer the conditions characteristic of forest fragments or favorable to dung and carrion beetle movements. Thus, in the fragmented landscapes of Los Tuxtlas, living fences that are biodiversity poor seem to be habitats that could make the agricultural matrix “biodiversity friendly” by acting as passageways for the fragments that are biodiversity rich (see Perfecto and Vandermeer 2008).

The structure of the landscape matrix in the buffer zone of the Los Tuxtlas Biosphere Reserve is important for the conservation of the communities of dung and carrion beetle species living in the remnant patches of forest. In the structure of these fragmented landscapes, it is desirable to avoid creating hard edges between forest fragments as these reduce the opportunities for beetles to cross pastures in search of other forest fragments. Living fences that connect fragments allow beetles to move among the fragments and favor gene flow among the populations inhabiting each fragment. Wider living fences would probably be even more favorable to beetle movement. Further studies will determine the corridor width that is appropriate for dung and carrion beetles, as well as for other species, allowing them to move among the forest fragments in the buffer zone and to connect with the nucleus zones in the reserve.
